# The effect of endocrine-disrupting chemicals on placental development

**DOI:** 10.3389/fendo.2023.1059854

**Published:** 2023-02-21

**Authors:** Yan Yan, Fengjun Guo, Kexin Liu, Rixin Ding, Yichao Wang

**Affiliations:** ^1^ Department of Endocrinology, The Second Hospital of Jilin University, Changchun, China; ^2^ Department of Obstetrics and Gynecology, The Second Hospital of Jilin University, Changchun, China; ^3^ Department of Gastrointestinal Colorectal Surgery, China-Japan Union Hospital of Jilin University, Changchun, China; ^4^ Department of Cardiovascular Medicine, Changchun Central Hospital, Changchun, China

**Keywords:** endocrine disrupting chemicals, placental development, bisphenol A, DDT, heavy metals, VU filters

## Abstract

Endocrine-disrupting chemicals (EDCs) or endocrine disruptors are substances that are either naturally occurring or artificial and are released into the natural environment. Humans are exposed to EDCs through ingestion, inhalation, and skin contact. Many everyday household items, such as plastic bottles and containers, the liners of metal food cans, detergents, flame retardants, food, gadgets, cosmetics, and pesticides, contain endocrine disruptors. Each hormone has a unique chemical makeup and structural attributes. The way that endocrine hormones connect to receptors is described as a “lock and key” mechanism, with each hormone serving as the key (lock). This mechanism is enabled by the complementary shape of receptors to their hormone, which allows the hormone to activate the receptors. EDCs are described as exogenous chemicals or compounds that have a negative impact on organisms’ health by interacting with the functioning of the endocrine system. EDCs are associated with cancer, cardiovascular risk, behavioural disorders, autoimmune abnormalities, and reproductive disorders. EDCs exposure in humans is highly harmful during critical life stages. Nonetheless, the effect of EDCs on the placenta is often underestimated. The placenta is especially sensitive to EDCs due to its abundance of hormone receptors. In this review, we evaluated the most recent data on the effects of EDCs on placental development and function, including heavy metals, plasticizers, pesticides, flame retardants, UV filters and preservatives. The EDCs under evaluation have evidence from human biomonitoring and are found in nature. Additionally, this study indicates important knowledge gaps that will direct future research on the topic.

## 1 Introduction

Endocrine-disrupting chemicals (EDCs) are exogenous compounds that can interact with the endocrine system in humans, altering critical biological processes such as immunity, metabolism, organogenesis, reproduction, and behavior. Several synthetic and natural chemicals can disrupt the endocrine system’s function and negatively impact the body. These compounds are known as EDCs (1). Natural or artificial substances can act as hormone disruptors. Other than these pesticides, heavy metals, and retardants, everyday products like plasticizers (food containers, flame retardants, plastic bottles, cosmetics, detergents, and toys) expose people to EDCs. Some EDCs, such as polychlorinated biphenyls (PCBs), dichlorodiphenyldichloroethylene (DDE), dioxin, organochlorine pesticides, and hexachlorobenzene (HCB), were designed to have long half-lives for industrial purposes and are known as “persistent organic pollutants” or POPs. These EDCs impede normal growth and development by interfering with genetic and epigenetic regulatory processes (2, 3).

The Environmental Protection Agency (EPA, USA) identified more than 86,000 substances as endocrine disruptors through the Toxic Substances Control Act. The effect of exposures increases during pregnancy since they may have an impact on the developing fetus and result in chronic postnatal diseases. In general, pregnant women experience rapid hormonal changes. If hormone production and secretion are interrupted, routine pregnancy maintenance and fetus nourishment become difficult (4). EDCs exposure increases a fetus’s lifetime risk of developing cognitive, behavioural, and reproductive abnormalities. According to reports, prenatal exposure to EDCs can cause disorders like cancer, autism, diabetes, infertility, and attention deficit hyperactivity disorder (5).

Immature fetuses are vulnerable to EDCs exposure because different EDCs are passed from the mother to the fetus through the placenta. Concerns regarding the contradictory effects of EDCs passed through the placenta during fetal growth have raised interest in the proper use of personal care products during placental development. Despite this, the placenta is affected by EDCs, preventing it from carrying out its normal activities. The direct effects of EDCs on the fetus are less well understood than their impact on placenta growth and function (6).

The placenta is a vital organ that performs a variety of endocrine, immunological, and physiological processes throughout pregnancy. The placenta gradually grows during the first three months of pregnancy, but starting in the fourth month, it develops simultaneously with the uterus. It is a transient organ that shares genetic characteristics with a developing infant (7). The placenta of the human body is a hemomonochorial type. It develops from a blastocyst early in gestation (trophectoderm layer). The trophoblast, which originates from the blastocyst’s outer layer, interacts with the decidual epithelium. Cytotrophoblasts (CTBs), syncytiotrophoblasts (STBs), and extravillous trophoblasts (EVTs) are the 3 primary trophoblast lineages. Recent research suggests that in the first trimester of pregnancy, EVTs invade the entire uterine vasculature, including arteries, veins, and lymphatics (8). The blastocyst’s trophectoderm cells differentiate to develop into the CTBs during placentation (first trophoblast lineage). The CTBs continue to multiply and differentiate into the STB layer and the EVTs. The uterine spiral arteries that provide oxygenated blood to the developing placenta and fetus are found in the placental bed. Throughout pregnancy, the placental bed undergoes significant modifications. Extravillous trophoblast (EVT) cells multiply from anchoring chorionic villi and invade the decidualized endometrium by two different pathways: (1) endovascular EVT cells invade the lumen of the spiral arteries, and (2) interstitial EVT cells invade the decidualized endometrium and inner myometrium. Some EVT are incorporated as intramural trophoblast into the spiral artery wall (9).

The lacunae or spaces are formed inside the syncytioblast, which are filled with maternal blood as it comes into contact with blood vessels. Lacunar networks are formed when these lacunae join together. The maternal blood that passes into and out of these networks exchanges nutrients and waste materials with the fetus, forming the foundation of uteroplacental circulation (10). The primary chorionic villi (PCV), which pierce and spread into the surrounding syncytiotrophoblast layer, originate from the cytotrophoblast layer. These structures are secondary chorionic villi (SCV) when the extraembryonic mesoderm layer forms a central mass of loose connective tissue within this PCV post-implantation. The SCV’s embryonic vasculature integrates with the embryonic mesoderm, forming tertiary chorionic villi (TCV). The syncytiotrophoblast secretes many proteins, including progesterone, human chorionic gonadotropin (HCG), and other substances (11).

**Figure 1:**
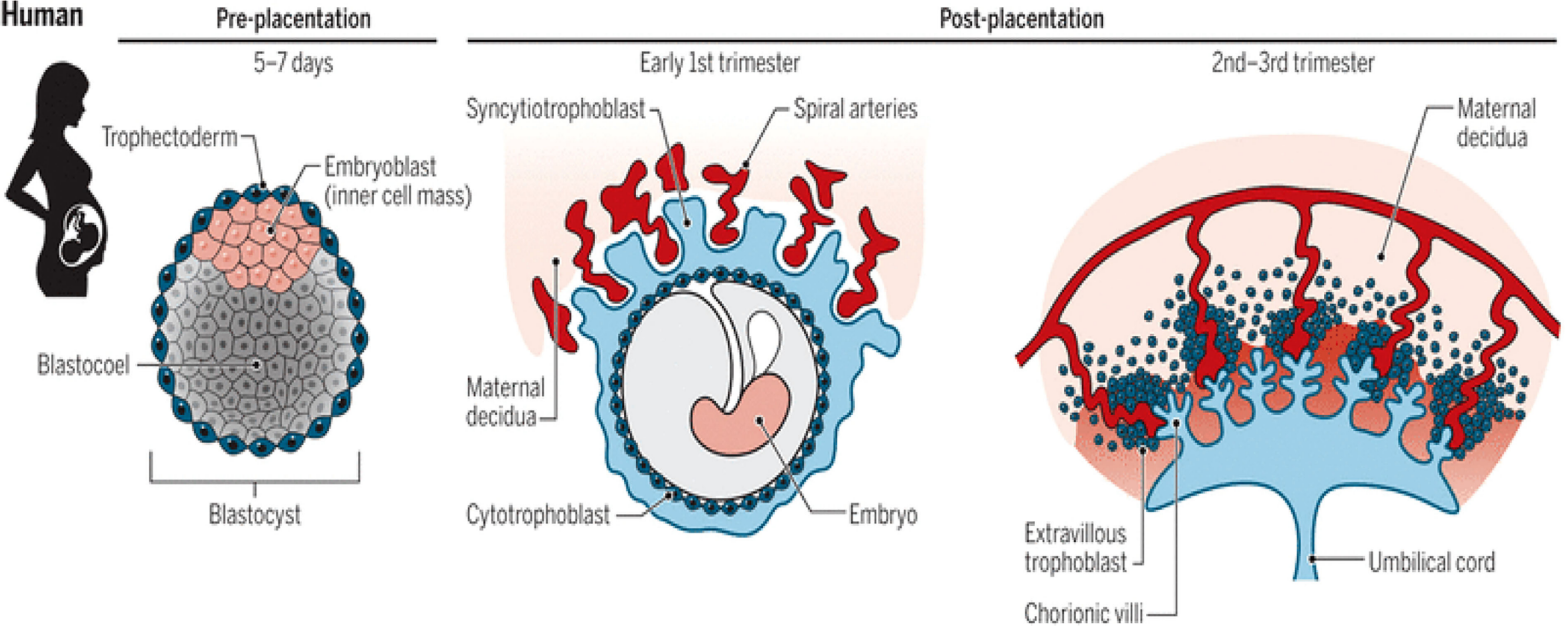
Stages (pre-and post-placentation) of placenta development (12).

To produce a cytotrophoblastic shell, the cytotrophoblast cells from the TCV advance toward the decidua basalis of the maternal uterus. Anchoring villi are the villi that attach to the decidua basalis *via* the cytotrophoblastic shell. Branching villi provide permeability for the interchange of metabolites between the fetal and maternal in the intervillous space by extending outward from the stem (anchoring) villi. Visualizing the villi as projections resembling trees can enable us to understand their anatomy (9). The mother’s spiral arteries undergo modification to develop low resistance and high blood flow conditions to fulfil the fetus’s needs. The mother’s spiral arteries are invaded by cytotrophoblast cells, which act as the endothelium’s replacement. They transform from epithelium to endothelium, expanding the diameter and decreasing the resistance of the blood vessels that supply the uterus with nutrient-rich blood (12). See **Figure 1**.

The placenta communicates with its surroundings. The placenta must develop for a pregnancy to be successful. This delicate organ comprises multiple layers of tissue that must develop normally during pregnancy to function correctly, which could have devastating consequences for the fetus (13). Different EDCs may indirectly influence the fetus through the placenta. Protein and hormone levels will fluctuate and balance as cells develop and differentiate. The hormonal environment will no longer be in equilibrium once EDCs have crossed the placenta and reached the fetus. Due to the immature fetus’ vulnerability to EDCs, preeclampsia (PE), fetal development retardation, miscarriage, and placental disruption might occur (2). Several genetic and epigenetic factors regulate the trophoblast layer’s proliferation, apoptosis, and invasiveness. Their imbalance hinders the early vascular system from generally developing in the first trimester of pregnancy, resulting in intrauterine growth retardation (14). The placenta is particularly susceptible to endocrine disruption because of the large density of steroid hormone receptor expression in this organ. Over the last five years, research on how EDCs impair placental development has grown significantly (15).

There are numerous constraints on how the EDC can be studied regarding placenta formation and development. As a result, this article examines how EDCs affect placental growth during the first trimester of pregnancy. We first discussed the several EDCs detected in the human placenta before revising how often these EDCs are exposed and how it affects the placenta and fetal maturation. We then discussed the mechanisms of action of dichlorodiphenyltrichloroethane (DDT), cadmium, and bisphenol-A (BPA), which are early-stage environmental EDCs that change placental characteristics.

### 2 Exposure of the placenta to endocrine-disrupting chemicals

Several EDCs have been studied because they can cross the placenta, accumulate in placental tissues, and persist in fetal circulatory systems and organs. Only a small percentage of the thousands of synthetic chemicals found so far have the potential to disrupt the endocrine system. Insecticides like DDT and dieldrin, flame retardants like polybrominated diphenyl ethers, fungicides, pharmaceuticals, and additives for consumer products (benzophenone, parabens, and synthetic musks like galactoside) are just a few examples of the numerous different types of EDCs (16). DDT exposure reduces placenta cell viability in vitro at doses more than 25 μM (HTR-8/SVneo), while smaller concentrations did not affect proliferation (1 nM). DDT (doses: 1, 10, or 100 ng/ml) enhanced the expression of prostaglandin E2 (PGE2) synthase, 3β-HSD, and CYP11A1 in bovine placental explants (17). Studies have shown that various EDCs are present in pregnant women’s urine, amniotic fluid, breast milk, and serum. EDCs can enter pregnant women through several pathways, including the respiratory system, packaging, the food chain, and the skin. Pregnant women occasionally breathe the dust from spaces with wallpaper or flooring, touch objects, or consume food or drink tainted with EDCs (18).

While the source of p,p′-DDE exposure is dependent on whether p,p′-DDT exposure is active, direct contact with pesticide application and nutrition are the main ways that humans are exposed to p,p′-DDT and o,p′-DDT. Humans convert p,p′-DDT to p,p′-DDE when exposed actively, but p,p′-DDE exposure is primarily acquired from the diet. Because p,p′-DDE has a longer half-life in the environment than its counterparts, it is more detectable and has higher levels in people, which leads to bio-accumulation in the food chain. Blood levels of p,p′-DDT, p,p′-DDE, and o,p′-DDT have dropped about five times since DDT was restricted in the early 1970s (19).

The placenta and fetus typically lack the enzymatic machinery to guard against these exposures. EDCs can disturb the endocrine system by competing with endogenous steroid hormones, binding to receptors and hormone transport proteins, or altering the breakdown or generation of endogenous hormones (20). Even though the mechanism of the protein responsible for regulating the transport of drugs in the placenta is only partially understood, more studies are needed to fully comprehend how EDCs move through the placenta and whether they can change gene expression. Over the past few years, numerous clinical investigations have been carried out to assess the level of EDCs in blood or amniotic fluid (21).

EDCs, such as the metabolites of phthalates, are linked to the polymer molecules in plastic products and can gradually leak into water or food packaged in plastic. Some of these might build up in placental tissues and affect how well the placenta functions. Urine samples were tested for triclocarban (TCC) and triclosan (TCS), substances found in a wide range of consumer goods, including toothpaste, plastics, textiles, medical equipment, and soaps. TCS was found in all study participants (22). Metabolites of parabens have long been employed as preservatives in numerous everyday items. Recent studies have shown that it functions as EDCs andis present in up to 100% (methylparaben=0.26 ng/mL, ethylparaben=0.40 ng/mL; isopropylparaben=0.18 ng/mL; *n*-propylparaben=0.18 ng/mL; iso-butylparaben=0.07 ng/mL; *n*-butylparaben=0.07 ng/mL; and benzylparaben=0.18 ng/mL, respectively) of the mother urine samples analyzed (23).

According to another research, organochlorine pesticides have also been related to pre-term birth, fetal cryptorchidism, and prenatal development retardation (24). Exposure to polychlorinated biphenyls and polybrominated diphenyl ethers during the prenatal period inhibits the development of the neurological system and is linked to fetal thyroid malfunction. The placental barrier was also breached by polycyclic aromatic hydrocarbons (PAH), phenols, perchlorate, and perfluorinated chemicals found in 98 to 100% of samples from expecting mothers (25). Since BPA exhibits estrogenic action, it has the potential to disrupt endocrine pathways. It has been established that the presence of BPA (1 nM) in trophoblast cells affects DNA methylation during the first trimester of pregnancy and inhibits cellular growth. Studies showed that BPA enters the placenta and increases the level of β-HCG, which affects placental development (26).

BPA has been found in maternal and fetal serum and the human placenta and can cross the placental barrier. As a result, BPA can enter the fluids and tissues of the human womb. BPA can also be absorbed by contact or inhalation. For instance, the receipts’ thermal paper may produce this substance when it comes into contact with the epidermis (27, 28). Recent research on metabolism and toxicokinetics has revealed that BPA is rapidly absorbed orally. Once ingested, this substance is coupled with glucuronic acid in the liver. BPA glucuronate is a reliable exposure biomarker and is adequately stable (29). Although some controversial data suggests that BPA is not hazardous to human health, several recent studies have highlighted its negative consequences. BPA can accumulate in various human and animal tissues due to its lipophilic nature (logP of 3.4), impairing their physiological processes and negatively impacting health (30, 31).

**Figure 2:**
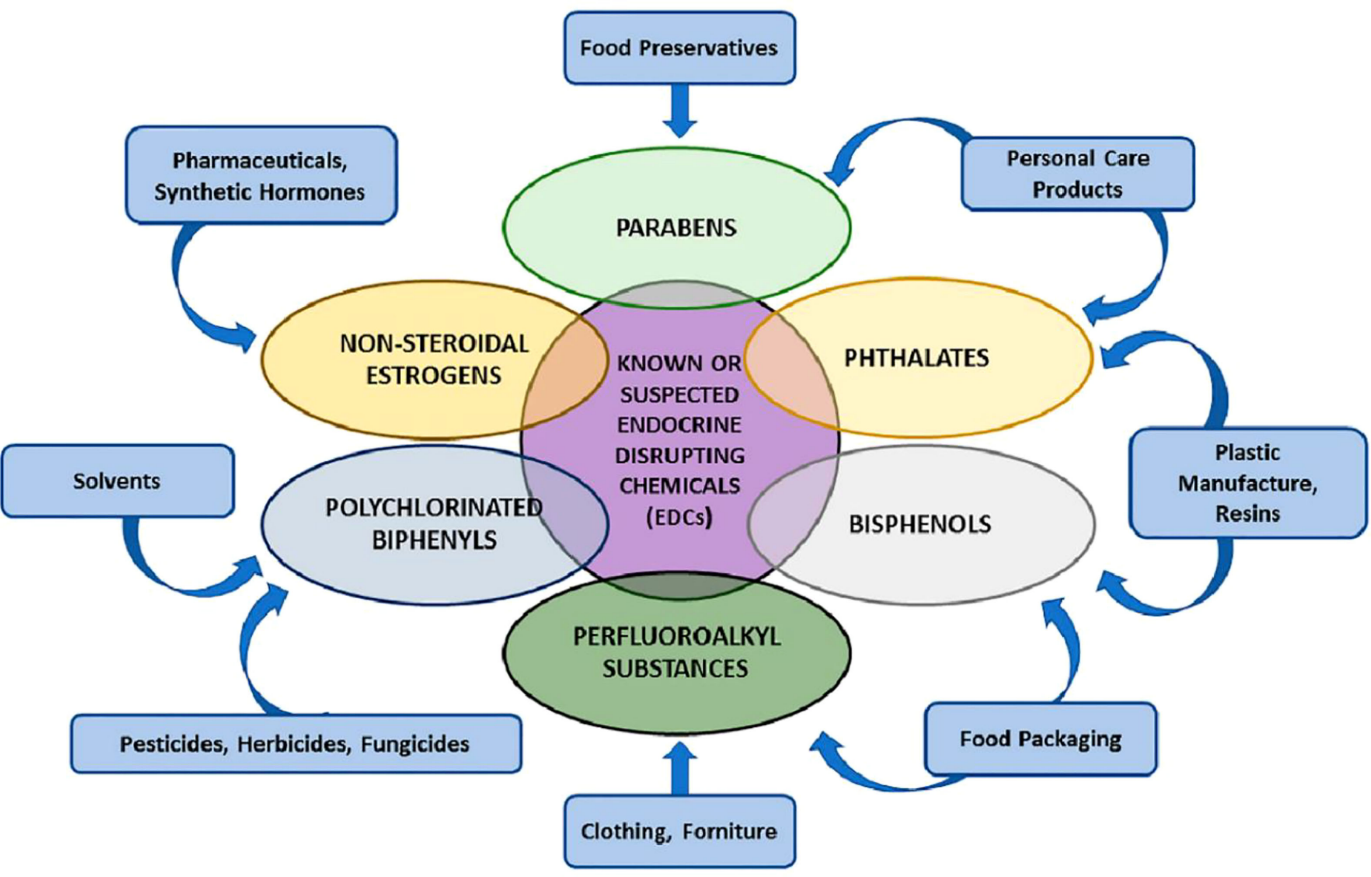
Various groups of endocrine-disrupting chemicals (EDCs) (34).

*IGF-2* gene methylation alterations are associated with exposure to various persistent organic pollutants (POPs), including DDTs. *IGF-2* is a crucial regulator of fetal and placental development (32). The amounts of heavy metals in placental tissues have been investigated using various techniques. These heavy metals include mercury (Hg = 550 µg/m^3^), lead (Pb = 500 mg/kg/day), and cadmium (Cd = 0.02 µg/g), all of which have been linked to brain disorders and abnormalities in vertebrates. Even though various hormone disruptors have been identified, few human endocrine routes have been thoroughly investigated, and little is known about the impacts of epigenetics. There are many unexplored pathways and components in normal physiology. New research and new research approaches are required for this field to increase knowledge of future human diseases (33). The estrogenic signaling pathways are disrupted by a class of substances known as xenoestrogens (PCBs, BPA, and phthalates). Initial xenoestrogen exposure (~3.24 ng/day) may also prevent trophoblast penetration of the endometrium, increasing the risk of intrauterine growth restriction (IUGR), *in-utero* neonatal mortality, and epigenetic abnormalities in the placenta for the infant (34) (**Figure 2**).

## 3 Impact of EDCs on placentation

### 3.1 Heavy metals

Although heavy metal pollutants’ effects on the fetus’s development have not been examined, this study can provide insight into how metal intakes result in these effects. Cd, Pb, Hg, and As have all been identified as worldwide pollutants by the Agency for Toxic Substances and Disease Registry (ATSDR, Atlanta, GA, USA) (35). These substances are toxic to human development and cross the placenta. These metals are exposed more frequently because they are used in many residential, agricultural, technical, and medical applications (36).

#### 3.1.1 Cadmium (Cd)

Cd is one of our environment’s most prevalent and dangerous heavy metals. Decades after its toxicity was discovered, research on its toxicity is still a popular topic in systematic works. The central nervous system, liver and kidney in the placenta and fetus as teratogenicity, as well as the *in-vivo* studies conducted on experimental models to assess the maternal cadmium-induced toxicity (alteration in essential element homeostasis, preeclampsia, kidney injury, bone mineralization, and fertility) were summarized (37).

Several studies have reported that Cd exposure during placentation reduces the levels of membrane proteins, decreasing their transport function and increasing intracellular Cd accumulation in the placenta of Cd-treated (5 mg/kg) rats. The histopathological studies showed that exposure to Cd causes swelling in trophoblast cells and vacuolar degeneration and prevents the migration of human trophoblast cells to the placenta. Long-time exposure induces apoptosis in syncytiotrophoblast cells in-vivo rate model (38). Given that each cigarette contains 1-2 µg of Cd, numerous analyses have demonstrated a link between Cd ingested through tobacco smoke and pregnancy problems. Its precise mode of action has also been discovered (39). Furthermore, during placenta development, Cd causes the discharge of volatile substances such as cytokines from the trophoblast cells of humans and the mouse placenta (40).

Moreover, Cd exposure during gestation in mother tissues such as maternal blood, mammary glands, and the placenta induces the production of metallothionein (MTs) in the syncytiotrophoblast and amniotic cells. The Cd-MT complexes will avoid the Cd transport from the placenta to the fetus. High MT (8-9 μg/g) will increase the formation of zinc-MT complexes. Cd may reduce the efficiency of Zn transport, such as ZnT2, ZIP14, and DMT1, and prevent the conveyance of Cd and Zn to the fetus. Even though the exact mechanism by which Cd moves through the placenta and into the fetus is still undetermined, research on rats conducted by Nakamura et al. suggests that DMT1 is the protein involved in the transport of Cd from the placenta to the fetus. On the other hand, it would appear that placental MTs would help prevent Cd transfer; however, the exact process is still unclear at this moment. According to Nakamura et al., the expression of MT genes and the level of Cd in the placental regulates the induction of metal transporter. This suggests that the mechanism of Cd mobilization and transport in the placenta depends on various metal proteins, including the MTs (41).

#### 3.1.2 Mercury (Hg)

Hg exposure is primarily caused by eating contaminated fish and seafood, which mainly builds up in the fetal CNS (central nervous system). Ask et al. analyzed the levels of inorganic mercury (I-Hg) and methylmercury associated with selenium in the placentas of pregnant women. Increasing concentration determined their accumulation in the placenta (42). Gilman et al. discovered that women who did not consume seafood had a higher proportion of Hg in the placenta to cord blood than those who consumed. Furthermore, the placenta contains more selenium than cord blood in this population (43). However, studies have shown that Mercuric Hg does not readily pass the placental barrier but accumulates in the placenta and amnion. Further studies need to evaluate the mechanism of impact of disruption in placentation (44).

#### 3.1.2 Lead (Pb)

Common causes of exposure to Pb include Pb-based paint and glazed food containers. Pb also causes the fetus to undergo apoptosis and interferes with the ability of organelles like the endoplasmic reticulum and mitochondria to operate appropriately. According to publications on how metals can affect trophoblast features, prenatal exposure to Cd (0.44–2.68 ng/g) or Pb (17.17–46.86 ng/g) can cause many illnesses, including obesity, by genetic and epigenetic regulation (45). There have been numerous studies on the cytotoxic effects of Pb on fetuses in development, but little is known about the mechanisms of action at low concentrations.

Heavy metals have been shown to interfere with protein kinase C (PKC) and Ca2^+^ binding proteins, disrupt Ca2^+^ channels and pumps, and impact calcium (Ca2^+^) homeostasis (CaBP). Despite the modest maternal and cord blood Pb levels, there was a strong correlation between maternal blood Pb concentration (13.35 ± 0.73 nmol) and a reduction in syncytiotrophoblast Ca2^+^ uptake. Arsenic (As) exposure generally happens when people consume contaminated water and make pesticides. On the other hand, Zinc (Zn) is a trace element that naturally occurs in the earth, air, and food (46).

### 3.2 Plasticizers

Chemicals created by humans called plasticizers are primarily employed to increase polymers’ elasticity, flexibility, color, resistance, and/or durability. The most extensively used plasticizers include phthalates, alkylphenols, and BPA. di-(2-ethylhexyl) phthalate (DEHP), butyl benzyl phthalate (BBP), dibutyl phthalate (DBP), and dimethyl phthalate (DMP) are a few of the most often used phthalates (47). Phthalates were categorized as EDCs in 2002 because they interfere with the secretion, binding, transport, synthesis, and/or removal of endogenous hormones from the body, disrupting hormonal systems. Because fetal and embryonic development is especially vulnerable, plasticizers have been shown to cross the human placenta and reach the fetus (48).

DEHP (2 mmol/L) exposure decreases cell proliferation. It reduces placenta size in mothers, which is associated with fetal growth limitation and adult disorders, according to the *in-vivo* and *in-vitro* investigation. DEHP inhibits placental cell proliferation, but the exact mechanism by which it does so is still unclear (49). Furthermore, several studies have indicated that DEHP may negatively affect fetal cellular mechanisms such as oxidative stress response, invasion, immunomodulation, endocrine function, and trophoblast differentiation, which may lead to pathologies and unfavourable pregnancy outcomes (50). Around 98–100% of pregnant women have had phthalate metabolites, including DEHP (1.7 μg/L) and MEP (monoethyl phthalate, 1.2 μg/L), found in their urine (51). Strong evidence suggests that prenatal phthalate exposure was linked to altered placental size and form. Exposure to phthalates (~3 to 30 μg/kg/d) could cause the placenta to become thicker and more circular rather than round or oval with a centrally inserted umbilical cord (52).

Numerous studies have identified a relationship between phthalate metabolites in human third-trimester urine samples and target genes involved in the trophoblast differentiation and steroidogenesis pathways. The manifestation of a gene involved in trophoblast differentiation was inversely correlated with the presence of phthalate metabolites, including; butylbenzyl phthalate (BBzP), diisobutyl phthalate (DiBP), di-n-butyl phthalate (DnBP), and DEHP (53). Additionally, mono(2-ethylhexyl) phthalate (MEHP) decreased extravillous trophoblast (EVT) invasiveness, a crucial component of placentation. In human trophoblast cells, BPA, nonylphenol (NP), and octylphenol (OP) similarly cause cytotoxicity (>500 μM) (54). According to studies, DEHP administration at GD 13 significantly reduced placenta weight. Others have found that the DEHP-treated placenta significantly decreased Esx1, Ascl2, and Fosl1 mRNA expression levels at GD 13. DEHP injection disrupted the labyrinth vascularization of the placenta, which also hampered development and induced apoptosis (55).

Numerous studies have shown that BPA exposure around the time of implantation has a negative impact on placentation and uterine renovation. The occurrence and size of placentas, fetuses, and implantations were studied in pregnant female mice exposed to 50 µg/kg BPA/day or 0.1% ethanol by oral gavage from day 1 to day 7 of gestation. Blood flow in the arteria uterine was also examined, and the results showed that the remodeling of the uterine spiral arteries (SAs) was severely compromised (56). There is strong evidence that BPA has been identified in follicular fluids, maternal, human fetal, and amniotic, as well as deposits found in placenta cells (57). In addition to blocking human trophoblast cell invasion, this chemical significantly modifies the structure of mouse placental layers and exhibits characteristics similar to those of PE (58). Recent studies indicate that BPA exposure may directly harm the placenta, leading to abnormal trophoblast cell labyrinthine development that negatively impacts trophoblast hCG production in the first trimester and elevates cell mortality. However, it has been found in multiple studies that BPA reduced HIF-1α levels and increased levels of anti-apoptotic proteins, including Hsp70 and Bcl-2 in BeWo cells, hence reducing apoptosis (59).

According to studies, BPA at low doses (1–1000 nM) could activate the ERK signaling pathway to suppress the expression of CYP11A1 and CYP19, lower placental aromatase activity, and reduce the generation of estradiol and progesterone (60). The usage of bisphenol S (BPS, 0.5 mg/kg/day), a BPA substitute, has also been linked to endocrine dysfunction and interference with trophoblast fusion in the placenta (61). It is worth noting that a neuroendocrine mechanism was hypothesized in some studies where BPA (62.5-25.0 mg/kg) exposure impacted pituitary function in infantile rats, lowering basal and GnRH-induced LH and increasing GnRH pulsatility (62).

### 3.3 Pesticides

Insect, weed, and fungal growth are all controlled by a class of organic molecules known as organochlorine pesticides (OCPs), which include carbon, hydrogen, and chlorine. According to statistics, 40% of all pesticides used in diverse pest control methods come from the chemical class of organochlorines. This broad category of chlorinated hydrocarbons includes DDT and its metabolites, hexachlorobenzene (HCB), lindane, and dieldrin. They can become active, enter the mother’s bloodstream, and eventually reach the placenta (63).

These substances can potentially impair fetal growth and the terminal stage of placental life by interfering with the placenta’s ability to produce and release hormones, and enzymes, transport nutrients, and produce waste. In the epidemiological model, high *p,p’*-DDT are substantially correlated with insulin-like growth factor 2 (*IGF2*), which may help explain fetal development restriction. Maternal organochlorine pesticide exposure levels (0.7-1.7 ng/g lw) were also linked to the hyper-methylation of placental genes (64).

OCP compound residues in the placenta of females are associated with pre-term and full-term deliveries. The glutathione and malondialdehyde were assessed as oxidative stress markers, and significantly higher levels of α-HCH (63.04 ng g^-1^), total HCH (41.5 ng/g lw), *p,p*-DDE (59.3 ng/g lw), and total DDT (5100 ng/g lipid) in the placenta were recorded (65). Similarly, in venous and umbilical cord blood from pregnant Hispanic women, biomarkers of OCPs were assessed. Low exposure to these OC chemicals during placental development may have impacted the development of the fetus. They may have caused later health issues like diabetes, obesity, cancer, and endocrine disruption (66). Another study discovered that various pesticides, such as methoxychlor (OC) and fenitrothion (OP), are human multi-resistant associate protein substrates (MRP1). MRP2 is expressed in the syncytiotrophoblast, whereas MRP1 and MRP3 are expressed in the blood vessel endothelia and the syncytiotrophoblast (67). Blood cholinesterase and tissue carboxylesterases (CEs), which are reliable markers, can determine whether an individual has been exposed to OPs. Robinson et al. (68) evaluate the results of OP exposure (median=28.5 nmol/L) on placental CE action and lipid composition in humans and hypothesize that these pesticides similarly enter the placenta. Lower CE function may have toxicological and clinical implications that can disrupt the growth and development of the fetus. Lipid profile changes suggest cytotrophoblast hyperplasia as a potential healing strategy.

In addition, the OCPs may act as endocrine disruptors leading to pre-term birth (PTB) through disturbance of the average estrogen-progesterone ratio. Significantly higher levels of α-hexa-chlor-ocyclohexane (α-HCH; 40 mg/kg/day), β-hexa-chlor-cyclohexane (β-HCH; 5 mg/kg/day), dichloro-diphenyl-dichloroethane (DDD; ≥5.83 ng/g lipid), and dichloro-diphenyl-dichloroethylene (DDE; 45.4–94.6 ng/g lipid) were found in maternal blood and placenta as well. Other studies indicate a risk of early exposure for babies because of chronic bioaccumulation and inadequate removal, which could have a negative impact on health (69). Several pesticides and their compounds can survive in the food chain due to their lipophilicity and resilience to environmental degradation (70).

### 3.4 Flame retardant

Numerous types of furniture and electronic devices have been treated with brominated flame retardants (BFRs), such as polybrominated diphenyl ethers (PBDEs) and 2,4,6-tribromophenol (2,4,6-TBP), which are also used as reactive flame retardants. A class of EDCs known as PBDEs has been extensively used in electronic equipment, construction materials, foam, textile materials, and automobile parts (71). Similar chemical structures are shared by 2,4,6-TBP and PBDEs, raising questions about how they might affect thyroid hormone (TH) regulation. Numerous epidemiological studies show that exposure to PBDE during placentation affects the regulation of THs by interfering with TH serum transporters, may affect TH concentrations in the placenta, and may affect TH delivery to the fetus. Although 2-, 4-, and 6-TBP are not well-studied, data show that human exposure is widespread and ongoing, similar to PBDE exposure (72). *In vitro* studies have shown that using a human primary villous cytotrophoblast (CTB) model demonstrates polybrominated diphenyl ethers (BDE-47) to inhibit the ability of human EVTs in the first trimester to migrate and invade (73).

Numerous research has investigated how PBDE exposures may cause placenta abnormalities. Greater maternal PBDE levels were strongly linked to worse newborn head circumference (HC) and Apgar1 scores (73). Multiple metabolic abnormalities may alter the connections by escalating oxidative stress, regulating vasodilation, mediating neurotoxicity, and causing dysbiosis in the mother’s gut microbiota (74). When discussing the potential link between PBDEs and neurodevelopmental issues, evidence for PBDE-induced epigenetic changes in DNA methylation, chromatin dynamics, and non-coding RNA expression was examined (75). At levels greater than 10 mM (BDE-47 and BDE-99), PBDE exposure is cytotoxic in first-trimester human EVTs, substantially lowering cell viability and inducing apoptosis. The same dose of BDE-47 also changed the metabolism of lipids and cholesterol while decreasing EVTs migration and invasion (76).

Fire-master 550 (FM 550) is a commercial flame retardant mixture (brominated and organophosphate) due to widespread human exposure and structural similarities with known EDCs. *In vivo*, the Wistar rat model indicated that these environmental chemicals might accumulate in the placenta, and there is evidence of an impact on sex-specific behavioral effects (77). Additionally, mothers’ exposure to these contaminants during pregnancy causes the bioaccumulation of flame retardant additives (FRAs) in their systems, including the placenta, blood, and serum. One of the exposure routes for FRAs is through human breast milk and blood or serum. These FRAs pollutants are transferred to infants through the mothers’ placenta, blood, and serum (69).

**Figure 3:**
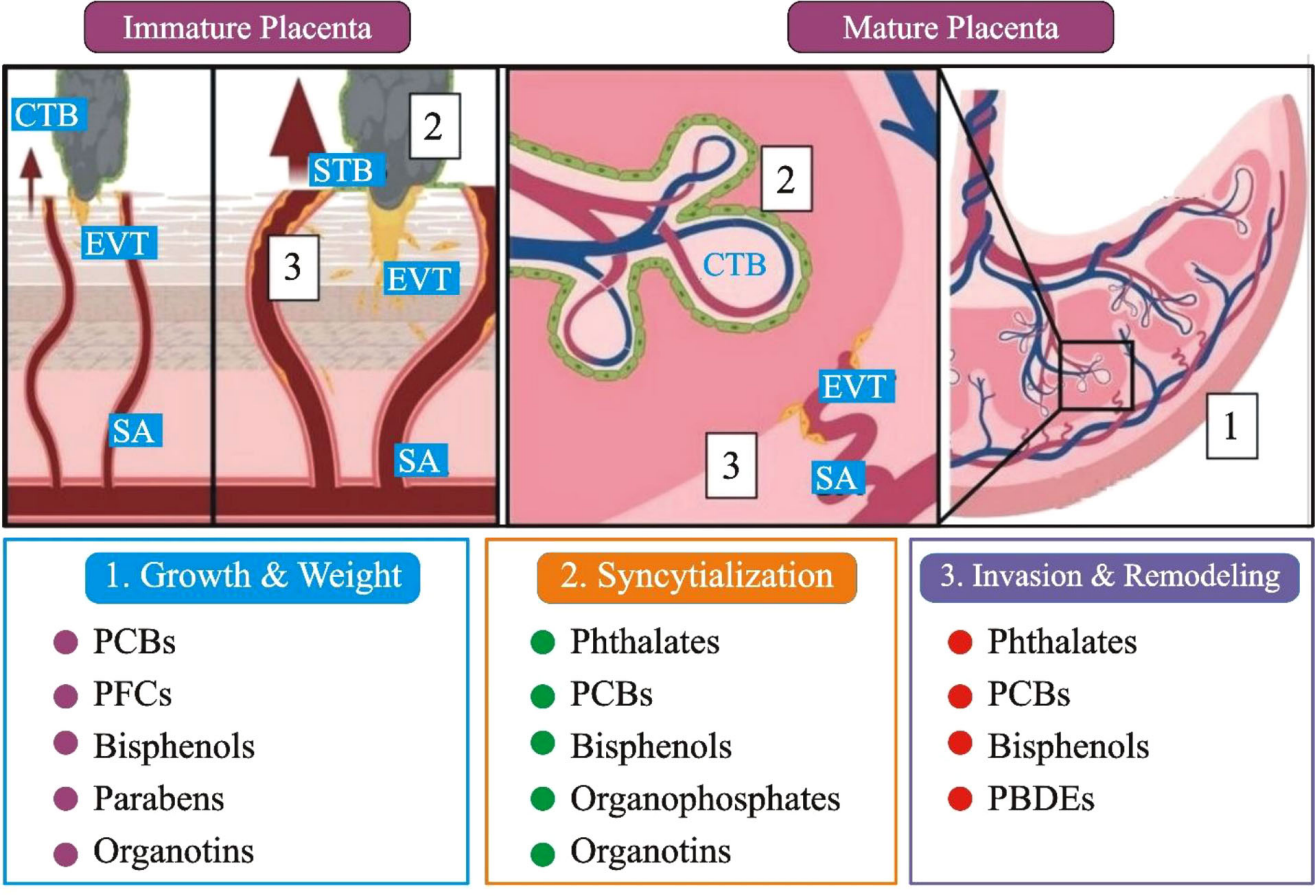
A comprehensive summary of the developmental, anatomical, and histopathological sites in the placenta that have been affected by EDCs exposure. (1). Immature placenta (left): cytotrophoblasts (CTB; grey) can differentiate into two separate lineages: invasive extravillous trophoblasts (EVT; yellow) and barrier syncytiotrophoblasts (STBs; green). The arrows represent that EVTs penetrate maternal tissues and, by modification of the spiral artery (SA), enable enhanced maternal blood flow to the placenta. (**2**). CTBs restore the STBs population in the mature placenta (right), while SAs that have undergone EVT remodeling fill the fetal villi in maternal blood. Placental gross mass/wet weight (grey box 1), CTB fusion/syncytialization (grey box 2), EVT invasion, and SA remodeling (grey box 3) are only a few of the changes caused by EDC; PCBs: polychlorinated biphenyls, PBDEs: polybrominated diphenyl ethers, and PFCs: perfluorinated compounds (**Figure 3** modified and edited from (79, 80)).

Other common chemicals used as flame retardants include organophosphate flame retardants (OPFRs) and perfluoroalkyl and polyfluoroalkyl substances (PFASs). PFAS and OPFRs were first studied in pregnant women’s mid-gestational urine samples. One of the morphological outcomes, along with potential placental stress markers, was the degree of uterine artery remodeling due to cytotrophoblast (CTB) (78). In addition, rodent studies suggested that PFASs (19.62 ng/ml) interfere with thyroid and placental function. These effects have been linked to various health effects, including bladder cancer, congenital hypothyroidism, and placenta-mediated adverse pregnancy and birth outcomes, such as preeclampsia, gestational diabetes, and low birth weight (**Figure 3**) (81).

The most often used brominated flame retardant for consumer products is tetra-Bromo-bisphenol A (TBBPA). The results of a study on the human first-trimester placental cell line HTR-8/SVneo show that TBBPA activates inflammatory pathways, specifically cytokine and prostaglandin synthesis. In HTR-8/SVneo cells, TBBPA increased interleukin (IL)-6, IL-8, and prostaglandin E2 (PGE2) release while suppressing TGF-β release (82). Similarly, TBBPA exposure (5, 10, 20, and 50 μM) was evaluated using placental explant cultures, which confirmed (at 50 μM) it induces placental inflammation, increases oxidative stress, and alters placental steroidogenesis (80). In vitro investigation revealed that TBBPA varies JEG-3 (choriocarcinoma-derived placental cells) estrogen synthesis due to its action on CYP19 protein expression. Thus this compound may interfere with normal placental development during the early stages (83).

### 3.5 UV filter and preservative

The UV filters and preservatives included in cosmetics, sunscreens, and personal care products have been the subject of several studies, but little is known about their potential effects on the placenta. Most of the antioxidants in the placenta belong to the paraben family, and concerns have been raised regarding how they can affect EDCs. One epidemiologic study focused on placenta-specific outcomes discovered a developmental abnormality in the placenta using a positive correlation between total maternal urinary paraben levels and placental weight (84). Pharmacokinetic research in pregnant rats is the only in vivo model that investigates gestational exposure; in this investigation, ethylparaben concentrations in the placenta (~3368-53,515 ng/g) and fetal liver were three times greater than in the fetal liver suggesting placental accumulation. In addition, the negative correlation between testosterone levels and ethylparaben concentrations in human cord blood suggests a possible danger to fetal development (85).

The movement and breakdown of the parabens ethyl, butyl, methyl, and propyl (EtP, BuP, MeP, and PrP), as well as their metabolite para-hydroxybenzoic acid (PHBA) *via* the human placenta, are demonstrated by an *ex vivo* human placental perfusion model (86). When excrement and amniotic fluid were tested for 2,4- and 2,5-dichlorophenols, bisphenol A, benzophenone-3, triclosan, and ethyl-, butyl-, and methyl and propyl, parabens, it was determined that amniotic fluid was positive for paraben (87).

It was shown through an in vitro investigation that butylparaben exposure reduces cell proliferation while inducing cell death and endoplasmic reticulum stress (200 μM) in HTR-8/SVneo cell lines (88) see **Table 1**. Apoptosis is triggered, and HTR8/SVneo cell proliferation is inhibited by the popular UV filter 4-MBC (95). Additional epidemiological studies have looked at the primary forms of parabens in pregnant women and their relationships with thyroid and reproductive hormones, suggesting alterations for methyl and butyl parabens (105). A few parabens are currently prohibited from cosmetics and cannot be used on newborns, toddlers, or children under three’s diaper area. Parabens, like methyl-, ethyl-, propyl and-butyl-parabens, are safe (106). Ultraviolet filters classified as EDCs with relation to the disruption of the hypothalamic-pituitary-gonadal (HPG) system include cinnamonate derivatives, camphor derivatives, and benzophenone (BP)-type. In several in vivo, in vitro, and in silico bioassays, BPs were found to have multiple endocrine-disrupting effects on the androgen receptor (AR), estrogen receptor (ER), progesterone receptor (PR), and other nuclear receptors (107).

**Table 1:** Worldwide *In vitro* and *In vivo* studies on EDCs exposure impact on placenta and placenta development.

EDCs	*In vitro* & *In vivo*	Experimental Model	Impact on Placentation	Authors
PBDE, OH-PBDE	*In vitro*	Trophoblast-derived cell line	Impaired thyroxine transport	89
BPA	*In vitro*	HTR-8/SVneo cell line	Proliferation and migration	90
BPA	*In vitro*	BeWo cell lines	Placenta (GPR30)	88
Firemaster 550	*In vivo*	Rat model	Placenta physiology	6
BPA	*In vivo*	Mouse model	Induce apoptosis in trophoblast cell	55
Homosalate	*In vitro*	HTR8/SVneo cell lines	Proliferation and invasiveness	91
BPA	*In vivo*	Mouse model	Spiral artery	53
BPA, BPS	*In vivo*	Pregnant sheep	Trophoblast signaling pathway	92
BPA, DEHP, PFOA	*In vitro*	Rhesus monkey TSC cell line	Trophoblast development and implantation	87
Methyl-Parabnan	*In vivo*	Pregnant mother	placental cholinergic function	93
PBDE	*In vivo*	Mouse model	Placental tissue signaling pathway	94
BPA, BPS	*In vitro*	Placenta tissue	Newborn recruited	95
BPA, Triclosan	*In vitro*	Human endometrial stomal cell line	Migration and decidualization	96
Phthalate, Phenol	*In vitro*	Pregnant women urine	Placenta (miRNA expression)	97
DEHP	*In vitro*	BeWo human trophoblast cell lines	Growth and invasiveness	98
Bifenthrin pesticide	*In vitro*	JEG-3 cell lines	Estrogen receptor pathway	99
Organochlorine	*In vitro*	Human trophoblast and MCF-7 cell line	Trophoblast proliferation and migration	100
Cadmium	*In vitro*	Human endometrial endothelial cell line	Endometrial angiogenesis	101
DDT	*In vivo*	Rat model	Induce apoptosis	102
Xenobiotic	*In vitro*	Trophoblast cell lines	Trophoblast invasion	103
DEHP	*In vivo*	Rat trophoblast cell line	Growth retardation	104
DDT, MTC	*In vitro*	JEG-3 human choriocarcinoma cell line	estrogen signaling pathways, placenta (Ca handling)	15

## 4 Conclusion and limitations of the study

This study highlights the current understanding of placental outcomes following EDC exposure by reviewing epidemiological *in vivo* and *in vitro* data. The use of supraphysiological dose regimens, which are often greater than human exposures and represent directly deleterious impacts, is one of the significant drawbacks. Human biomonitoring and *in vivo* pharmacokinetic data should be used to develop predictive physiologically-based toxicokinetic mathematical models to establish environmentally relevant dosage strategies. These doses should be used in conjunction with *in vitro* models that more closely replicate the *in vivo* placental microenvironments, such as 3-dimensional models that recreate cell-to-cell interactions and add tissue shear stress and extracellular matrix components. These alternative *in vitro* models should be utilized in conjunction with *in vivo* research incorporating suitable animal models to capture more human-representative toxicokinetic profiles or anatomical features. Unfortunately, the data on animal model utilization is lacking on this topic.

Finally, more research is needed to provide more exact effect estimates in risk assessment epidemiological investigations. Nonetheless, given the potential early impacts of endocrine disruptors on child health, pregnant women, infants, and young children should be given special care. This study also uncovers significant knowledge gaps that will guide future research.

## Author contributions

Study conception and design: YY and YW. Drafting of the manuscript: FG, KL, and RD. Critical revision: YY, FG and YW. Approve final version: All authors.

## Glossary

**Table:**

EDCs	endocrine-disrupting chemicals
EDs	endocrine disruptors
EPA	The Environmental Protection Agency
PCV	primary chorionic villi
TCV	tertiary chorionic villi
HCG	human chorionic gonadotropin
PE	preeclampsia
DDT	dichlorodiphenyltrichloroethane
BPA	phthalate-bisphenol-A
TCC	triclocarban
TCS	triclosan
PAH	polycyclic aromatic hydrocarbons)
BPA	bisphenol A
Hg	mercury
Pb	lead
Cd	cadmium
IUGR	intrauterine growth restriction
As	arsenic
MTs	Metallothionein
DEHP	Di-(2-Ethylhexyl)phthalate
BBP	butyl benzyl phthalate
DBP	dibutyl phthalate
MEP	monoethyl phthalate
NP	nonylphenol
OP	octylphenol
SAs	spiral arteries
BPS	bisphenol S
LH	luteinizing hormone
OCPs	organochlorine pesticides
DDT	Dichloro-diphenyl-trichloroethane
HCB	hexachlorobenzene
IGF2	insulin-like growth factor 2
MRP1	Multi resistant associate protein 1
CEs	carboxylesterases
PTB	pre-term birth
α-HCH	α-hexachlorocyclohexane
β-HCH	β-Hexa-Chlor-cyclohexane
DDD	dichloro-diphenyl-dichloroethane
DDE	dichloro-diphenyl-dichloroethylene
BFRs	brominated flame retardants
PBDEs	polybrominated diphenyl ethers
2, 4, 6-TBP	2, 4, 6-tribromophenol
CTB	Cytotrophoblast
FM-550	Fire-master 550
BDE-47	polybrominated diphenyl ethers 47
PFASs	polyfluoroalkyl substances
TBBPA	tetra-Bromo-bisphenol A
IL	Interleukin
PGE2	prostaglandin E2
PHBA	para-hydroxybenzoic acid
HPG	hypothalamic-pituitary-gonadal system
AR	androgen receptor
ER	estrogen receptor
PR	progesterone receptor
